# Undifferentiated pleomorphic sarcoma of the prostate in a young man

**DOI:** 10.1002/iju5.12174

**Published:** 2020-06-17

**Authors:** Yuya Iwahashi, Nagahide Matsumura, Hiroki Kusumoto, Takashi Ozaki, Masatoshi Higuchi, Yasuo Kohjimoto, Isao Hara

**Affiliations:** ^1^ Department of Urology Kinan Hospital Tanabe Wakayama Japan; ^2^ Department of Pathology Kinan Hospital Tanabe Wakayama Japan; ^3^ Department of Urology Wakayama Medical University Wakayama Wakayama Japan

**Keywords:** prostate sarcoma, robot‐assisted radical prostatectomy, undifferentiated pleomorphic sarcoma

## Abstract

**Introduction:**

Prostate sarcoma is an extremely rare disease with a poor prognosis. Undifferentiated pleomorphic sarcoma has never been described in the prostate.

**Case presentation**

A 27‐year‐old man complained of frequent urination and dysuria for several years. Various examinations were suggestive of prostate sarcoma. The pathological diagnosis was confirmed as prostate sarcoma via ultrasound‐guided transrectal needle biopsy. Because the location of the tumor in the prostate was confirmed by magnetic resonance imaging, we performed robot‐assisted radical prostatectomy. The final pathological diagnosis was undifferentiated pleomorphic sarcoma. Local recurrence occurred at the front of the rectum 2 months after surgery. Although chemotherapy and radiotherapy were initially effective, he died 18 months after surgery.

**Conclusion:**

Undifferentiated pleomorphic sarcoma of the prostate is believed to have a poor prognosis. When selecting the surgical procedure, functionality should be considered for individual cases with complete resection.

**Keynote message**

UPS of the prostate is an extremely rare disease. Despite its rarity, prostate malignancy must be considered when examining even young patients.

**Abbreviations & Acronyms**
MFH: malignant fibrous histiocytoma
MRI: magnetic resonance imaging
NR: not reported
OS: overall survival
UPS: undifferentiated pleomorphic sarcoma
US: undifferentiated/unclassified sarcoma

## Introduction

Prostate sarcoma is a rare disease accounting for 0.1–0.7% of all cases of prostate cancer.^1^ It occurs in adults aged 37–50 years old, which is much younger than the mean age at prostate cancer diagnosis. Leiomyosarcoma is reported to be the most common histology.^2^, ^3^ The chief complaint is often lower urinary tract obstruction because of prostate enlargement. Although the standard treatment is surgical excision, its prognosis is extremely poor when metastasis is found.

We report the case of a 27‐year‐old man who underwent robot‐assisted radical prostatectomy for prostate sarcoma. The pathological diagnosis was UPS. Local recurrence was observed after surgery. Complete response was achieved via the combination of chemotherapy and radiotherapy. Local recurrence occurred again, and he died because of multiple liver and lung metastases 18 months after the operation. To the best of our knowledge, this is the first report of UPS in the prostate.

## Case presentation

**Fig. 1:**
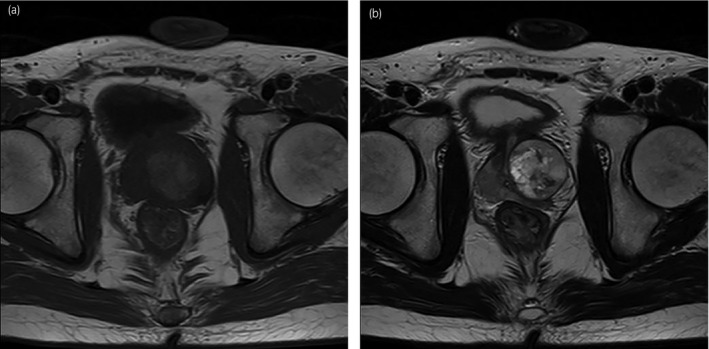
Magnetic resonance imaging (a: T1‐weighted image, b: T2‐weighted image) revealed a 4.6‐cm mass with heterogeneous high signal intensity.

A 27‐year‐old man with a previous history of anxiety neurosis complained of frequent urination and dysuria for a few years. The patient visited another urology hospital but received no treatment because the symptoms were believed to be caused by anxiety neurosis. He was admitted to our hospital because his symptoms gradually worsened. Digital rectal examination revealed a large stone‐hard palpable mass in left lobe. The patient’s serum prostate‐specific antigen level was 1.4 ng/mL (normal, ≤4 ng/mL), and another tumor marker was normal. Ultrasound revealed irregular swelling of the left lobe. MRI uncovered a 4.6‐cm mass with no sign of invasion to the prostatic fascia and seminal vesicles (Fig. 1). Further investigations including positron emission tomography computed tomography and bone scintigraphy were negative for metastasis. Ultrasound‐guided transrectal needle biopsy revealed a high‐grade tumor composed of round cells and spindle cells. Immunohistochemically, the tumor was positive for vimentin but negative for epithelial markers (CAM5.2, AE1/3, p63, and 34βE12). These results led to the diagnosis of prostate sarcoma. For treatment, we recommended surgical resection. Total pelvic exenteration could represent overtreatment because MRI revealed no sign of rectal invasion. We recommended total cystectomy, but the patient strongly wanted to preserve his bladder. We determined that complete excision was possible by expanding the resection to the bladder neck, and radical prostatectomy was finally performed.

**Fig. 2:**
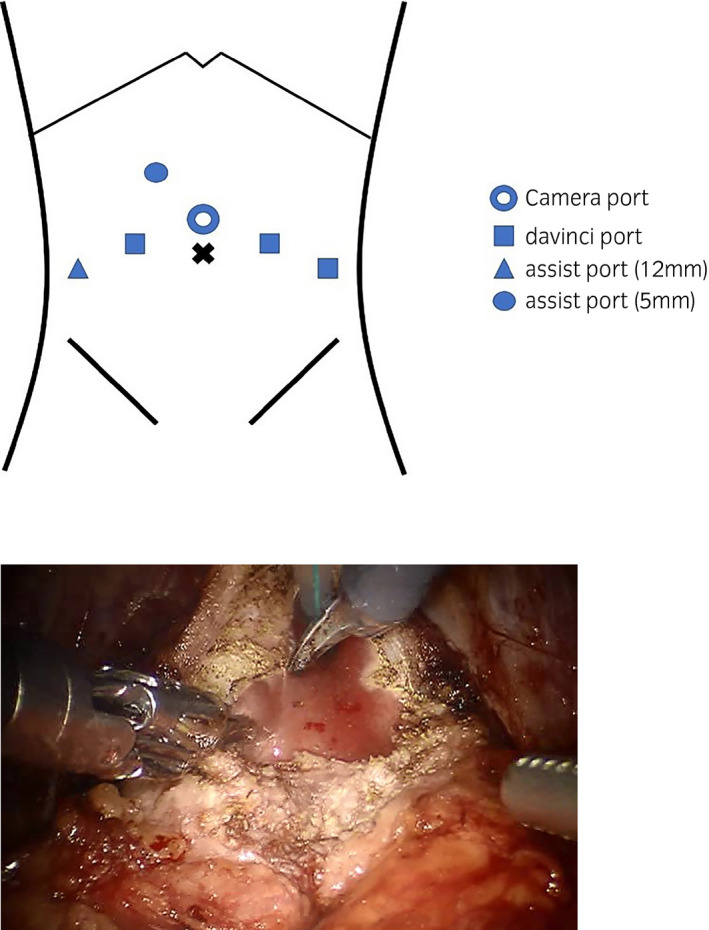
(a) Port placement in robot‐assisted laparoscopic radical prostatectomy. (b) Intraoperative picture when expanding the resection to the bladder neck.

Robot‐assisted laparoscopic radical prostatectomy was performed with extended pelvic lymph node dissection, sparing the right neurovascular bundle (Fig. 2). The prostate was removed without incision into the tumor by expanding the resection of the bladder neck as planned.

**Fig. 3:**
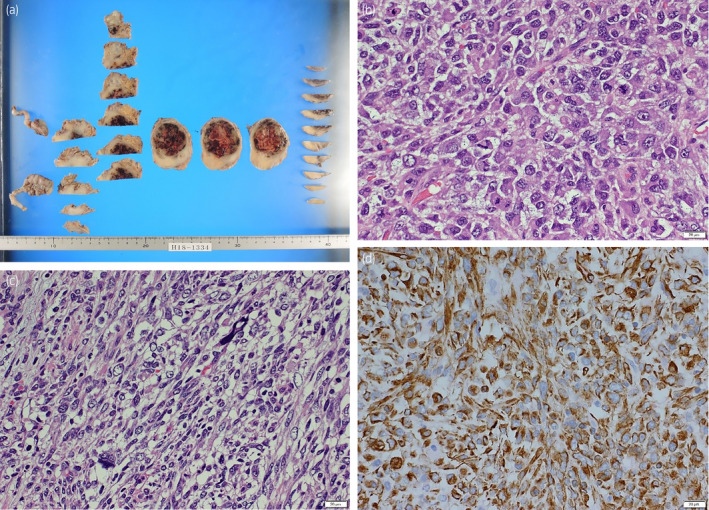
(a) Macroscopic findings revealed extraprostatic extension, but the resected margin was negative. (b) Atypical polygonal cells with enlarged nuclei were seen (HE, ×400). (c) Atypical spindle cells were seen (HE, ×400). (d) Immunohistochemical stains of vimentin was positive (vimentin ×400).

The operative time, console time, blood loss, and excised weight were 289 min, 242 min, 100 ml, and 110 g, respectively. The histopathological findings revealed spindle cells and round cells with nuclear atypia, but some of the cells exhibited showed various features such as striated muscle and smooth muscle. The result of immunostaining was identical to that of the prostate biopsy. Ultimately, the diagnosis was UPS arising from the prostate (Fig. 3). Although extraprostatic extension was positive, the resected margin was negative, and there were no metastatic lesions in the resected lymph nodes. Because there was no consensus of the effectiveness of adjuvant therapy against prostate sarcoma, no additional therapy was administered. However, computed tomography revealed recurrence at the front of the rectum 2 months after surgery, and we decided to administer chemotherapy and radiotherapy. Chemotherapy consisted of ifosfamide and doxorubicin (doxorubicin 30 mg/m^2^ × 2 days, ifosfamide 2 g/m^2^ × 5 days every 4 weeks) as the standard treatment for soft tissue sarcoma. Radiation (2 Gy × 28 times) was performed in combination with chemotherapy of third course. After three cycles of chemotherapy, a disease reevaluation confirmed a complete response; thus, we stopped chemotherapy after four cycles. Unfortunately, the tumor again recurred at the front of the rectum 8 months after surgery. We recommended same chemotherapy at the time of recurrence (8 months after surgery) since we achieved complete response once with chemotherapy consisted of doxorubicin and ifosfamide. However, he refused to undergo chemotherapy because he had adverse events such as nausea and febrile neutropenia during the previous chemotherapy. Therefore, we selected pazopanib. Although the patient was treated with pazopanib (800 mg/day), pazopanib was ineffective and multiple liver and lung metastases appeared and he died 18 months after the operation.

## Discussion

UPS was previously classified as MFH, but MFH was removed from the 2013 WHO classification. “US” represents tumors that cannot be classified into any other category because of the lack of distinguishing histological, genetic, or immunohistochemical features. UPS was recategorized as a subtype of US.^4^ Some cases of MFH in the prostate have been reported, but this is the first report of UPS in the prostate. UPS comprises approximately 5% of adult soft tissue tumors, most frequently arising in the extremities (68%), followed by the abdominal and retroperitoneum (16%) in patients aged 50–70 years old.^4^, ^5^ UPS is diagnosed histopathologically as the appearance of polymorphic cells and multinucleated giant cells. Immunohistochemically, the tumor cells were positive for vimentin but negative for several other markers. In addition, because various histological features are present within the same tumor, diagnosis is often difficult using a small amount of biopsy tissue, such as that obtained via needle biopsy.^6^ UPS invasively grows in surrounding tissues, and local recurrence is likely. Therefore, surgical resection with negative margins is essential. Table 1 shows the reported treatment outcomes of prostate sarcoma.^2^, ^3^, ^7^, ^8^ Although radical prostatectomy and cystoprostatectomy are chosen more frequently than total pelvic exenteration, there are no reports comparing prognosis between surgical approaches. Despite total pelvic exenteration, some patients experience recurrence and require multidisciplinary therapy. Therefore, when we select the surgical approach, functionality should be considered for individual cases with complete resection. In our case, the patient was 27 years old, and we selected radical prostatectomy with expanding resection of the bladder neck in consideration of functionality. He underwent multidisciplinary therapy because of recurrence, but the symptoms of frequent urination and dysuria were improved and functionality was preserved until immediately before death. As a treatment for recurrence, total pelvic exenteration was also considered, but the patient refused. There is no consensus of regimen for prostate sarcoma, and chemotherapy commonly used in soft tissue sarcoma are usually selected. Doxorubicin alone and doxorubicin and ifosfamide are chosen, and some cases have been reported in which chemotherapy are effective. However, Wang *et al.* reported that chemotherapy did not translate into improved OS.^3^ In fact, our case demonstrated complete response by doxorubicin and ifosfamide, which could not contribute to prolongation of survival time. In this case, a correct diagnosis was not obtained for some time because the prior urologist judged that the patient’s complaints were attributable to mental problems such as anxiety neurosis. Although it was extremely rare, we should consider the possibility of prostate malignancy when we examine even young patients with persistent urinary tract symptoms.

**Table 1:** Summary of studies reporting surgical approaches for prostate sarcoma

Author	No. of patients	Age† (years)	OS (months)	Surgical approach			
				Prostatectomy	Cystoprostatectomy	Total pelvic exenteration	Others
Bari (2017)	61	64.4	53	26	22	0	13
Musser (2014)	38	50	NR	6	13	8	11
Wang (2013)	25	37	23	2	10	2	11
Sexton (2001)	21	49	50	2	10	2	7

† Median age.

## Conflict of interest

The authors declare no conflict of interest.
